# Differential impact of substrates on myosin heavy and light chain expression in human stem cell-derived cardiomyocytes at single-cell level

**DOI:** 10.1007/s10974-025-09690-2

**Published:** 2025-02-13

**Authors:** Felix Osten, Alea K. Bodenschatz, Karina Ivaskevica, Simon Kröhn, Birgit Piep, Tim Holler, Jana Teske, Judith Montag, Bogdan Iorga, Natalie Weber, Robert Zweigerdt, Theresia Kraft, Joachim D. Meissner

**Affiliations:** 1 Institute of Molecular and Cell Physiology, Hannover Medical School, Hannover, Germany; 2 Leibniz Research Laboratories for Biotechnology and Artificial Organs (LEBAO), Department of Cardiothoracic, Transplantation and Vascular Surgery, Hannover Medical School, Hannover, Germany; 3 Department of Analytical Chemistry and Physical Chemistry, Faculty of Chemistry, University of Bucharest, Bucharest, Romania; 4 Present Address: Department of Nuclear Medicine, Hannover Medical School, Hannover, Germany; 5 Present Address: Faculty of Medicine, MSB Medical School Berlin, Berlin, Germany; 6 Present Address: Institute of Molecular and Translational Therapeutic Strategies (IMTTS), Hannover Medical School, Hannover, Germany

**Keywords:** Embryonic stem cell-derived cardiomyocytes, Myosin heavy chain, Myosin light chain, Culture substrate, Stiffness

## Abstract

To fully exploit the potential of human pluripotent stem cell-derived cardiomyocytes, ideally they should acquire a mature, adult ventricular-like phenotype. Predominant expression of the β-isoform of myosin heavy chain (β-MyHC) and the ventricular isoform of myosin regulatory light chain 2 (MLC2v) is a marker of human adult cardiac ventricle. Yet predominant co-expression of these isoforms is rarely reported by current culture protocols. Here, we assessed the impact of different substrates on β-MyHC and MLC2v expression in single human embryonic stem cell-derived CMs (hESC-CMs). As substrates, surface materials with differing stiffness as defined by Young’s modulus were combined with either laminin, a single-component coating, or Matrigel, a multi-component coating including growth factors. Semi-quantitative single-cell immunofluorescence analysis demonstrated that surfaces with supraphysiological stiffness in combination with laminin are sufficient for promotion of predominant β-MyHC expression, but not for predominant MLC2v expression in hESC-CMs. Accordingly, mechanical stimuli likely promote expression of β-MyHC in these cultures. Culture on matrices with a lower stiffness than glass in combination with growth factor-containing Matrigel led to only moderate increases in MLC2v expression, possibly more dependent on growth factors, suggesting different regulation of expression. Integrin-related downstream signal transducers, integrin-linked and cardiac troponin I-interacting kinase, as well as modulation of intracellular Ca^2+^-concentration and epigenetic signaling did not affect MyHC/MLC2 isoform expression. The data indicate that expression of adult ventricular markers β-MyHC and MLC2v depends on different stimuli like substrate stiffness and growth factors. To conclude, multiple stimuli appear to be necessary to promote an adult ventricular phenotype.

**Supplementary Information:**

The online version contains supplementary material available at 10.1007/s10974-025-09690-2.

## Introduction

Human pluripotent stem cell-derived cardiomyocytes (hPSC-CMs) have great potential for the study of cardiac biology and disease, and for therapeutic approaches (Yang et al. 2014), for example as a cellular model for hypertrophic cardiomyopathy (HCM). To fully exploit this potential, hPSC-CMs ideally should acquire a mature, adult ventricular-like phenotype. Predominant expression of the β-isoform of myosin heavy chain (β-MyHC) and expression of the ventricular isoform of myosin regulatory light chain 2 (MLC2v) is a marker of adult cardiac ventricle, while α-MyHC is predominantly and MLC2a is only expressed in human atria (Reiser et al. 2001; Sheikh et al. 2015). Many different stimuli have been employed to improve a comprehensive maturation of hPSC-CMs (Jiang et al. 2018), yet current culture protocols do not reliably report quantitative single-cell data on predominant co-expression of the ventricular myosin isoforms β-MyHC and MLC2v. Notably, the mature state of human cardiomyocytes is not solely determined by β-MyHC and MLC2v but also by expression of other sarcomeric (e.g. *TNNI3* for cardiac troponin I, cTnI) and non-sarcomeric genes as well as by morphological and functional characteristics (Jiang et al. 2018). Indeed, some differences in cross-bridge-related mechanical parameters were found in subcellular myofibrils within hESC-CMs compared with ventricular myofibrils isolated from adult donor hearts (Iorga et al. 2018).

The function of extracellular matrix (ECM) is far from only providing a passive scaffold for cells and tissues (Rozario and DeSimone 2010). The ECM contributes to key biological processes such as development, growth, and differentiation. For example, the ECM has important structural and functional roles for cellular function and tissue architecture in the heart (Del Monte-Nieto et al. 2020). Substrate stiffness has been shown to affect maturation of hPSC-CMs (Ribeiro et al. 2015; Jiang et al. 2018). We have previously demonstrated that long-term culture on a stiff matrix, laminin-coated glass coverslips, a 2D culture model, increased the number of exclusively β-MyHC expressing human embryonic stem cell-derived cardiomyocytes (hESC-CMs) compared with culture as cardiac bodies (CB) in suspension, a 3D culture model representing a soft matrix (Weber et al. 2016). Furthermore, substrate stiffness has been shown to influence cell size (Herron et al. 2016), and modulating substrate stiffness has profound effects on hPSC-CM function in 2D as well as in 3D culture (Denning et al. 2016; Guo and Pu 2020).

Changes in ECM stiffness are detected by mechanosensation and transmitted into the cell by mechanotransduction, leading to cardiomyocyte remodeling under physiological and pathophysiological conditions (Saucerman et al. 2019). Several mechanosensation/-transduction-related pathways have been shown to affect cardiomyocyte function (Dostal et al. 2014). Integrins are transmembrane receptors involved in mechanosensation/-transduction of changes in ECM stiffness (Pentassuglia and Sawyer 2013). We have recently demonstrated that integrin downstream mediator focal adhesion kinase (FAK) promoted expression of β-MyHC but not of MLC2v on stiff matrix (Osten et al. 2023), indicating that the expression of these markers for mature cardiomyocytes is differentially regulated.

Integrin-linked kinase (ILK) is a heart-enriched kinase and scaffold protein also acting downstream of integrins, suggesting a role in mechanotransduction (Hannigan et al. 1996). ILK was implicated in cardiomyogenesis and cardiac differentiation (Traister et al. 2012; Zhang et al. 2022), suggesting ILK to be another interesting candidate for regulation of MyHC expression. The same may apply to cardiac troponin I-interacting kinase (TNNI3K), a cardiac-specific kinase with sequence similarity to ILK that has been shown to promote mouse ESC-differentiation into cardiomyocytes (Wang et al. 2017; Zhao et al. 2003).

Epigenetic modifications constitute a level of gene regulation acting far downstream of the cell membrane in signaling cascades, leading either to gene activation, e.g. by acetylation of histones, or inhibition, e.g. by methylation of DNA (Rothbart and Strahl 2014). p300 is a transcriptional co-activator with histone acetyltransferase (HAT) activity that can additionally acetylate non-histone proteins (Dancy and Cole 2015). β-MyHC in cardiac and MyHCI in skeletal muscle are encoded by the same gene (Lompre et al. 1984). We have previously demonstrated that p300 is involved in Ca^2+^/calcineurin-dependent activation of a *MYH7* promoter in mouse skeletal muscle C2C12 myotubes (Meissner et al. 2011). *MYH7* gene expression is repressed by methylation of CpG islands in its promoter region via DNA-methyltransferase 3A (DNMT3A) (Fang et al. 2016). Repression of *MYH7* gene transcription by CpG-methylation can be reversed by 2-oxoglutarate (α-ketoglutarate)-dependent dioxygenase ten-eleven translocase 2 (TET2), that catalyzes the conversion of 5-methylcytosine (5mC) to 5-hydroxylmethycytosine (5hmC), thereby constituting the first step in cytosine demethylation (Loenarz and Schofield 2008; Iyer et al. 2009; Tahiliani et al. 2009; Wu and Zhang 2017).

Here, we assessed the impact of different substrates (surface and matrix combinations) and signaling pathway modulation on β-MyHC and MLC2v expression in hESC-CMs at single-cell level. The data suggest that expression of adult ventricular markers β-MyHC and MLC2v in hESC-CMs depends on different stimuli like substrate stiffness and growth factors.

## Materials and methods

### Cell culture

hESCs were differentiated into CMs from a hES3 αMyHCneoPGKhygro cell line as cardiac aggregates in suspension culture based on previously described protocols (Schwanke et al. 2014; Weber et al. 2016; Osten et al. 2023; Kriedemann et al. 2024). hESC-CM aggregates were dissociated on day 12–15 of differentiation with Collagenase B (1 mg/mL; Roche) in low calcium solution (Maltsev et al. 1994) for 30 min at 37 °C under gentle agitation or alternatively with StemDiff kit (STEMCELL Technologies) in accordance with manufacturer’s instructions. Dissociated CMs were resuspended in IMDM Glutamax (Thermo Fisher Scientific) supplemented with 10% fetal calf serum (GE Healthcare Life Science) and 10 µM Rho-associated protein kinase (ROCK) inhibitor Y-27632 (Tocris) and the cell suspension was seeded onto laminin-coated (Merck; 0.02 mg/mL in PBS) glass coverslips (standard conditions). Alternatively, cells were seeded onto laminin- or Matrigel-coated (Corning; 9.73 µL/mL dissolved in DMEM/F12, Thermo Fisher Scientific) glass, Aclar (polychlorotrifluoroethylene; Plano), or polydimethylsiloxane (PDMS; Specialty Manufacturing Inc.) coverslips. Aclar and PDMS were supplied as film sheets and die-cut into 18 or 32 mm diameter coverslips, followed by sterilization with 80% ethanol for 15 min and finally washed with PBS before use. After 24 h, medium was switched to basic serum-free (bSF) medium (high glucose, pyruvate, no glutamine Dulbecco’s modified Eagle’s medium supplemented with 2 mmol/L L-glutamine, 1% non-essential amino acids, 100 U/mL penicillin/streptomycin, 0.1 mmol/L β-mercaptoethanol; all Thermo Fisher Scientific, 17 μg/mL sodium selenite, 11 μg/mL transferrin, and 10 μg/mL human insulin; all Sigma). For the first 7 days in adherent culture, media was additionally supplemented with 200 µg/mL G418 (Geneticin; Thermo Fisher Scientific) to enrich for CMs, as the αMyHCneoPGKhygro line carries a selection marker for Neomycin controlled by the CM-specific α-MyHC promoter (Schwanke et al. 2014). hESC-CMs were cultured up to 35 days on coverslips and medium was changed twice per week (every 3–4 days).

### Stiffness of culture surface materials

Young’s modulus E (stress/strain) of Aclar (polychlorotrifluoroethylene) film and of polydimethylsiloxane (PDMS) film was measured as a determinant of stiffness using a 6800 Series Universal Testing System (Instron, Norwood, MA, USA) at Niedersächsisches Zentrum für Biomedizintechnik (NIFE, Hannover, Germany).

### Ca^2+^ ionophore treatment

hESC-CMs cultured on laminin-coated glass coverslips for 7 days were treated with 0.01 µM, 0.02 µM, or 0.04 µM Ca^2+^ ionophore A23187 (Sigma) dissolved in DMSO (Sigma; 0.2 mg/mL stock solution) for 2, 3, and 4 days.

### Inhibitor treatments

hESC-CMs cultured on laminin-coated glass coverslips for 7 days were treated with 0.5 or 2 μM ILK inhibitor Cpd22 (Calbiochem), 25 or 500 nM TNNI3K inhibitor GSK114 (AOBIOUS), or 1 or 5 μM p300 inhibitor C646 (Calbiochem) for 3 days. All compounds were dissolved in DMSO (1 mM stock solutions).

### Dimethyl 2-oxoglutarate treatment

hESC-CMs cultured on laminin-coated glass coverslips for 7 days were treated with 4 mM membrane permeable 2-oxoglutarate (α-ketoglutarate) derivate dimethyl 2-oxoglutarate (Sigma) for 2 and 4 days. For treatment duration, medium was changed every 24 h.

### β1 integrin antibody treatment

hESC-CMs cultured for 7 days on laminin-coated glass coverslips were treated with β1 integrin activating antibody (1:100; MAB1951Z, Merck) for 4 days. For treatment duration, medium was changed every 24 h.

### Semiquantitative single-cell immunofluorescence (IF) analysis

After culture for indicated number of days, cells were fixed in 4% paraformaldehyde (PFA; Merck) for 1 h and stored in PBS at 4 °C. For staining, coverslips were first washed with PBS and then incubated in 0.2% Triton X-100 (Roche; diluted in PBS) for 15 min for permeabilization. Alternatively, 5 µm thick cryosections from human interventricular septum or atrium embedded in Tissue-Tek O.C.T. Compound (Sakura) and obtained with a Leica CM1860 UV cryostat were incubated in 0.5% Triton X-100 for 30 min after fixation and washing. Coverslips were washed twice with PBS and blocked in 5% bovine serum albumin (BSA; Gerbu) dissolved in PBS (blocking solution) for 20 min. Primary antibodies against α-MyHC (rabbit, polyclonal, α-huMYH6, BioGenes), β-MyHC (mouse, monoclonal, M8421, Sigma-Aldrich), MLC2a (mouse, monoclonal, 311 011, Synaptic Systems), MLC2v (rabbit, polyclonal, 10906-1-AP, Proteintech), proBNP (mouse, monoclonal, ab13115, abcam), α-actinin (rabbit, monoclonal, ab68167, abcam), and p300 (mouse, monoclonal, sc-48343, Santa Cruz) were incubated in blocking solution for 1 h. Coverslips were washed twice with PBS and incubated with secondary antibody Alexa 488 donkey anti-rabbit (A21206, Thermo Fisher Scientific) and Alexa 555 donkey anti-mouse (A31570, Thermo Fisher Scientific) in blocking solution for 1 h. Coverslips were washed once and then nuclei stained with 4′, 6-diamidin-2-phenylindol (DAPI; Sigma; diluted 1:12,500 in PBS) for 5 min. After a final PBS and ddH_2_O washing step, coverslips were mounted with Fluoroshield mounting medium (Sigma) on glass slides. Images were captured using an Olympus IX51 or Olympus IX83 inverted fluorescence microscope.

#### MyHC/MLC2 analysis

Single-cell IF analysis of MyHC and MLC2 isoform expression was performed as described previously (Weber et al. 2016, 2020; Osten et al. 2023). In brief, cells were stained with specific antibodies either against α- and β-MyHC, or against MLC2a and MLC2v, and individual cells in each raw image file were analyzed for signal intensity of isoforms based on sarcomere staining only. CMs were then classified and scored according to MyHC or MLC2 isoform signal intensity, respectively, as described previously (Weber et al. 2020; Osten et al. 2023), and depicted in Figures accordingly.

#### MyHC isoforms

Red: β, exclusively β-MyHC expressing CMs; orange: β > α, co-expression of α- and β-MyHC with higher level of β-MyHC fluorescence; yellow: α = β, co-expression of α- and β-MyHC with equivalent fluorescence levels; α > β: light green, co-expression of α- and β-MyHC with higher level of α-MyHC fluorescence; green: α, exclusively α-MyHC expressing CMs.

#### MLC2 isoforms

Magenta: MLC2v, exclusively MLC2v expressing CMs; purple: v > a, co-expression of MLC2v and MLC2a with higher level of MLC2v fluorescence; dark blue: v = a, co-expression of MLC2v and MLC2a with equivalent fluorescence levels; a > v: light blue, co-expression of MLC2v and MLC2a with higher level of MLC2a fluorescence; cyan: MLC2a, exclusively MLC2a expressing CMs.

#### proBNP analysis

hESC-CMs were cultured for 14 and 35 days and stained against proBNP and cardiac troponin T or α-actinin as sarcomeric markers. CMs from 30 images per condition, captured using a 40 × objective, were analyzed for proBNP signal intensity using ImageJ 1.53t (National Institutes of Health, USA). The expected proBNP-containing perinuclear region (Pohjolainen et al. 2020), identified by proBNP staining and nuclear counterstain with DAPI, was selected using the ‘Freehand selection’ tool in ImageJ. The manually selected region of interest was then analyzed using the ‘Measure’ function to obtain mean pixel density (mean gray value).

### Western blots

Following culture for 9 and 11 days, hESC-CMs were lysed in kinase assay lysis buffer (20 mM Tris–acetate, pH 7.0; 0,1 mM EDTA; 1 mM EGTA; 1 mM Na_3_VO_4_; 10 mM β-glycerolphosphate; 50 mM NaF; 5 mM pyrophosphate; 1% Triton X-100; 2 µg/mL leupeptin; 0.27 M sucrose, supplemented with Protease Inhibitor Cocktail (Bimake) and Phosstop Phosphatase Inhibitor Cocktail (Roche)). Alternatively, tissue samples of human interventricular septum were prepared using the same lysis buffer. Prepared samples were then mixed 4:1 with ROTI®Load 1 4 × concentration (Carl Roth) and loaded onto 10% SDS gels for separation of proteins by SDS-PAGE. Using a wet tank transfer system (Bio-Rad), proteins were transferred onto a nitrocellulose blotting membrane and subsequently blocked in 5% milk powder dissolved in Tris-buffered saline (TBS) with 0.1% Tween 20 (Sigma). Membranes were incubated with primary antibodies against ILK (rabbit, monoclonal, 3856, New England Biolabs), TNNI3K (rabbit, polyclonal, SAB21025, Sigma), GAPDH (mouse, monoclonal, AKR-001, BioCat), FAK (mouse, monoclonal, sc-271126, Santa Cruz), phospho-FAK (rabbit, polyclonal, 3283, Cell Signaling), ERK1/2 (rabbit, polyclonal, 9102, Cell Signaling), phospho-ERK1/2 (rabbit, polyclonal, 9101, Cell Signaling), and β-tubulin (mouse, monoclonal, G098, Applied Biological Materials) at 4 °C overnight on a shaker. Incubation with HRP-conjugated secondary antibodies against mouse (from goat, 1721011, Bio-Rad) and rabbit (from goat, 1706515, Bio-Rad) was done for 1 h at RT. Western blot images were acquired with an ImageQuant LAS4000 imaging system using enhanced chemiluminiscence (SuperSignal™ West Dura Extended Duration Substrate).

### Morphological analysis

Cellular morphology was assessed by evaluating parameters including cell area, aspect ratio, and myofibrillar alignment. For this analysis, microscopic images (20 × objective) of cells stained for β-MyHC as described above were utilized. Only cells with β-MyHC staining of sarcomeres were used for morphological analyses. Cell area was measured using the ‘polygon selection’ tool and aspect ratio was measured using the ‘straight line’ tool with subsequent ‘Measure’ function in ImageJ software. The aspect ratio was determined as the ratio between the maximum cell length and maximum cell width (length-to-width ratio). Myofibrillar alignment was quantified using the FibrilTool plugin in ImageJ (Boudaoud et al. 2014). To improve the precision of alignment score calculations, a bandpass filter was applied to the images to suppress sarcomeric striation patterns of myofibrils prior to analysis.

### Ingenuity pathway analysis (IPA, Qiagen)

For analysis of NCBI gene datasets of *MYH6* and *MYH7* (MyHC-related genes) or *MYL2* and *MYL7* (MLC2-related genes), a list of associated genes was obtained by entering the search queries “*MYH6*”, “*MYH7*”, “*MYL2*”, and “*MYL7*” on https://www.ncbi.nlm.nih.gov/gene (accessed 27 March, 2024) and limiting to Homo sapiens. The resulting dataset of 160 MyHC-related genes or 53 MLC2-related genes without duplicates was downloaded as a text file and imported into Ingenuity Pathway Analysis (IPA) using the Core Analysis function. Default settings were used for the analysis, with confidence of prediction restricted to experimentally observed values only and the selected species restricted to humans only, and the reference set limited to Ingenuity Knowledge Base genes only. The resulting data was ranked based on the p-value of overlap as calculated by right-tailed Fisher’s exact test.

### Statistical analysis

For plotting of data and for statistical analysis, GraphPad Prism 9.5.1 was used. For statistical analysis of two groups, an unpaired Student’s t-test was used. Statistical differences between three or more groups were analyzed using one-way ANOVA with Tukey’s multiple comparison test to compare the mean of each group with the mean of every other group, or with Šídák’s multiple comparison test to compare the means of preselected pairs of groups (day 7 vs. day 10). The p-value of overlap in Ingenuity Pathway Analysis was based on right-tailed Fisher’s exact test. Significant differences are designated as p < 0.05 (*), p < 0.01 (**), p < 0.001 (***), or p < 0.0001 (****).

## Results

### Substrate-dependent effects on MyHC and MLC2 isoform expression in single hESC-CMs

**Fig. 1:**
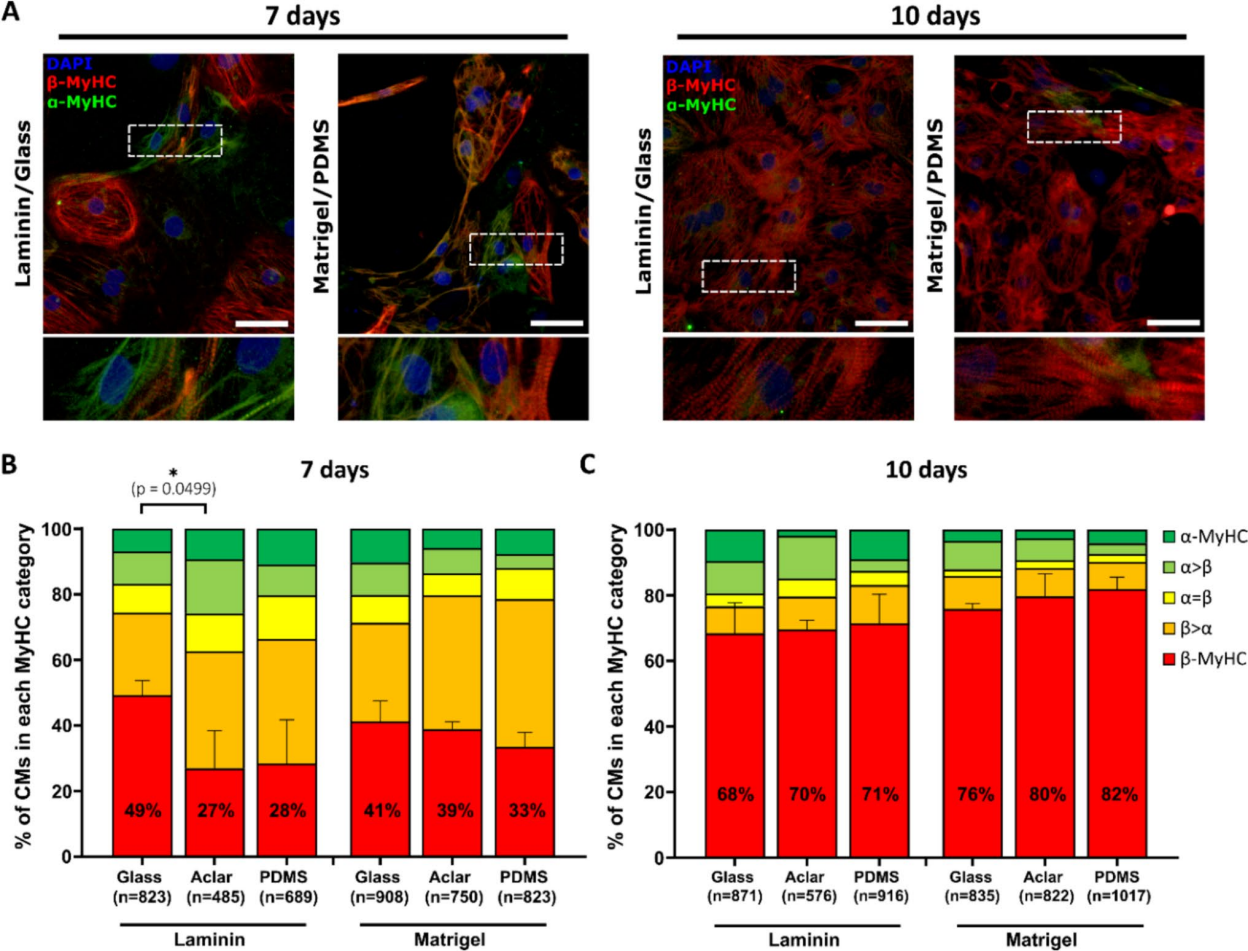
Analysis of MyHC isoform expression in single hESC-CMs cultured on different substrates (surface and matrix combinations). **A** Immunofluorescence (IF) analysis of MyHC isoform expression in hESC-CMs cultured on laminin-coated glass coverslips or Matrigel-coated PDMS for 7 or 10 days by using specific antibodies against α- (green) and β- (red) MyHC as indicated. Representative IF images. Nuclei were stained with DAPI (blue). Bottom: Magnified views of indicated regions showing sarcomeric striations as basis of classification. Scale bars: 50 μm. **B**, **C** Semiquantitative IF analysis of single hESC-CMs cultured on laminin- or Matrigel-coated glass, Aclar, and PDMS coverslips for 7 (**B**) or 10 days (**C**). The fractions of cells in the different categories (see Material and methods) are shown as percentage of the total number of cells analyzed (n, set to 100%). CMs from 3 coverslips of one differentiation. Mean ± SD. p-values are derived from one-way ANOVA with Tukey’s multiple comparisons test (see Tables 1, S1) of exclusively β-MyHC expressing CMs (red) only. *p < 0.05

**Fig. 2:**
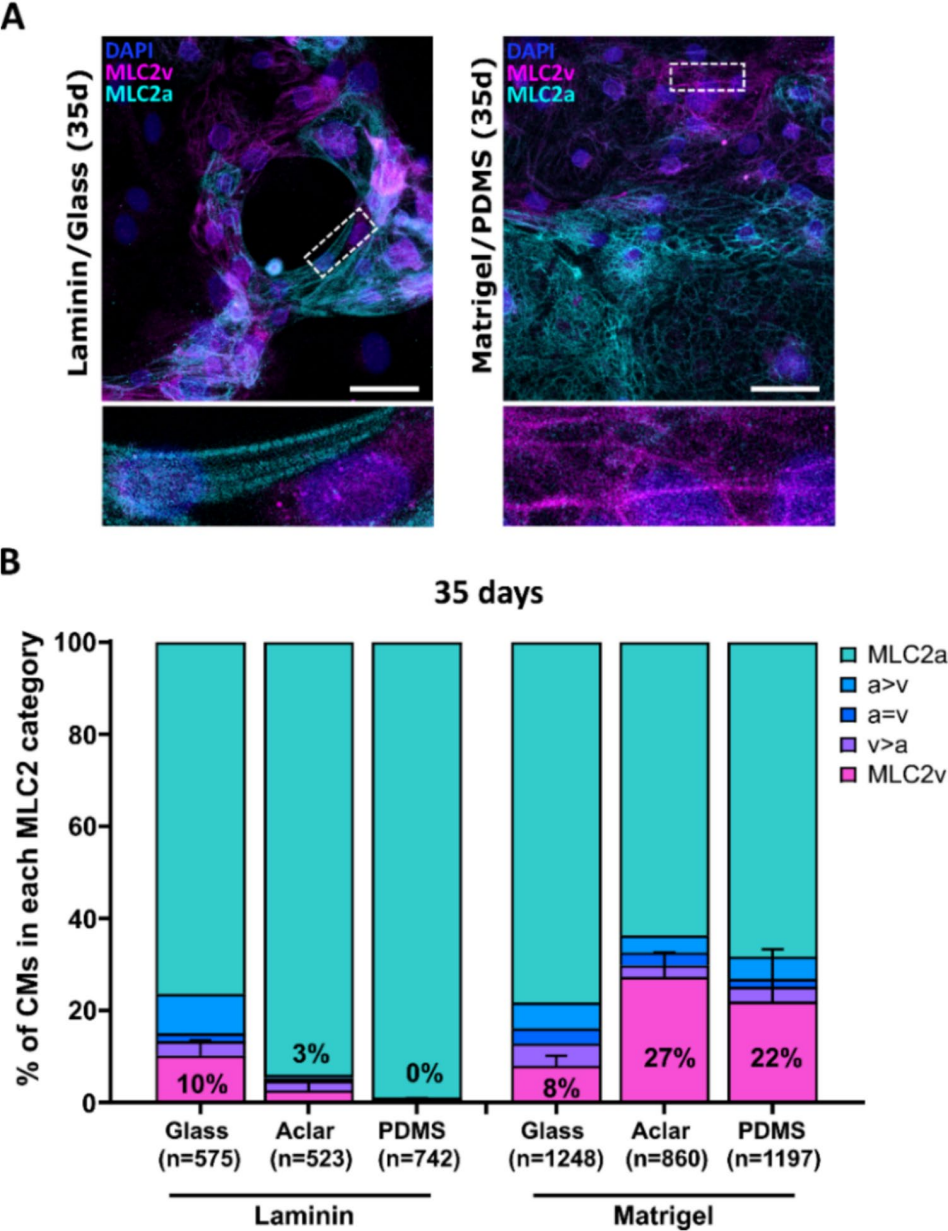
Analysis of MLC2 isoform expression in single hESC-CMs cultured on different substrates (surface and matrix combinations). **A** IF analysis of MLC2 isoform expression in hESC-CMs cultured on laminin-coated glass coverslips or Matrigel-coated PDMS for 35 days by using specific antibodies against MLC2a (cyan) and MLC2v (magenta). Representative IF images. Nuclei were stained with DAPI (blue). Bottom: Magnified views of indicated regions showing sarcomeric striations as basis of classification. Scale bars: 50 μm. **B** Semiquantitative IF analysis of single hESC-CMs cultured on laminin- or Matrigel-coated glass, Aclar, and PDMS coverslips for 35 days. The fractions of cells in the different categories (see Material and methods) are shown as percentage of the total number of cells analyzed (n, set to 100%). CMs from 3 coverslips of one differentiation, except for Matrigel-coated glass and PDMS (5 coverslips of two differentiations). Mean ± SD. p-values are derived from one-way ANOVA with Tukey’s multiple comparisons test of exclusively MLC2v expressing CMs (magenta) only (see Table 2)

We have previously shown that changes in substrate stiffness have a profound impact on MyHC isoform expression in hESC-CMs (Weber et al. 2016). To investigate the impact of substrate stiffness on the expression of MyHC and MLC2 isoforms in single hESC-CMs in more detail, coverslip materials with differing stiffness (Aclar, glass, PDMS) were combined with either a single-component coating (laminin) or a multi-component coating containing growth factors (Matrigel). From all culture surface materials used, glass coverslips have the highest (E = 85 GPa; (Seal et al. 2001)) and PDMS film the lowest (E = 742 kPa) stiffness, with Aclar film in between (E = 262 MPa). Expression of MyHC and MLC2 isoforms was analyzed at single-cell level using immunofluorescence (IF) analysis (Figs. 1A, 2A). After 7 days of culture, the substrate combination glass coverslips coated with laminin showed the highest proportion of exclusively β-MyHC expressing cells but was only significantly higher than the combination laminin on Aclar (Fig. 1B, Table S1). A significant increase in exclusively β-MyHC expressing cells after 10 days compared with day 7 was observed for all substrates, with no significant differences among substrates on day 10 (Fig. 1C, Tables 1, S1). Therefore, a limited effect of different substrates on β-MyHC isoform expression was only evident in early times of culture (7 days).

**Table 1:** Analysis of MyHC isoform expression in single hESC-CMs cultured on different substrates (surface and matrix combinations) for 10 vs. 7 days

Šídák’s multiple comparisons test	Adjusted p-value	Summary
Laminin/Glass d7 vs. Laminin/Glass d10	0.0203	*
Laminin/PDMS d7 vs. Laminin/PDMS d10	< 0.0001	****
Laminin/Aclar d7 vs. Laminin/Aclar d10	< 0.0001	****
Matrigel/Glass d7 vs. Matrigel/Glass d10	< 0.0001	****
Matrigel/PDMS d7 vs. Matrigel/PDMS d10	< 0.0001	****
Matrigel/Aclar d7 vs. Matrigel/Aclar d10	< 0.0001	****

Overview of one-way ANOVA results with Šídák’s multiple comparisons test of data presented in Fig. 1, based on exclusively β-MyHC expressing hESC-CMs

*p < 0.05; ****p < 0.0001

After 7 days of culture, hESC-CMs did not express MLC2v yet on different substrate combinations (Fig. S1). To promote maturation of hESC-CMs and achieve a higher baseline MLC2v expression, cells were grown for 35 days. With laminin coating, glass coverslips showed the highest percentage of exclusively MLC2v expressing hESC-CMs, but the percentage was small (10%) and the differences were not significant (Fig. 2B, Table 2.) Using Matrigel coating, Aclar and PDMS showed the highest percentage of exclusively MLC2v expressing cells (27 and 22%, resp.) of all substrate combinations, significantly higher than the respective laminin-coated combination and Matrigel-coated glass coverslips (Fig. 2B, Table 2). To conclude, Matrigel coating facilitates the expression of MLC2v on surfaces with lower stiffness than glass (Aclar, PDMS). Nevertheless, in contrast to MyHC isoform expression, even after 35 days of culture, exclusively the atrial isoform MLC2a expressing hESC-CMs predominate in all conditions.

**Table 2:** Analysis of MLC2 isoform expression in single hESC-CMs cultured on different substrates (surface and matrix combinations) for 35 days

Tukey’s multiple comparisons test	Adjusted p-value	Summary
Glass/Laminin vs. PDMS/Laminin	0.4097	ns
Glass/Laminin vs. Aclar/Laminin	0.6716	ns
Glass/Laminin vs. Glass/Matrigel	0.9964	ns
Glass/Laminin vs. PDMS/Matrigel	0.1599	ns
**Glass/Laminin vs. Aclar/Matrigel**	**0.0388**	*
PDMS/Laminin vs. Aclar/Laminin	0.9971	ns
PDMS/Laminin vs. Glass/Matrigel	0.5484	ns
**PDMS/Laminin vs. PDMS/Matrigel**	**0.0024**	**
**PDMS/Laminin vs. Aclar/Matrigel**	**0.0008**	***
Aclar/Laminin vs. Glass/Matrigel	0.8326	ns
**Aclar/Laminin vs. PDMS/Matrigel**	**0.0067**	**
**Aclar/Laminin vs. Aclar/Matrigel**	**0.002**	**
**Glass/Matrigel vs. PDMS/Matrigel**	**0.0279**	*
**Glass/Matrigel vs. Aclar/Matrigel**	**0.007**	**
PDMS/Matrigel vs. Aclar/Matrigel	0.8423	ns

Overview of one-way ANOVA results with Tukey’s multiple comparisons test of data presented in Fig. 2, based on exclusively MLC2v expressing hESC-CMs

*ns* not significant

Significant results are highlighted in bold letters

*p < 0.05; **p < 0.01; ***p < 0.001

Assuming that mechanical stimuli likely promote expression of β-MyHC in hESC-CMs, we investigated the expression of proBNP, a marker of mechanical stress that is known to be synthesized by atria and ventricles upon increased cellular mechanical stimulation (Liang et al. 2000; Bhalla et al. 2004; Vanderheyden et al. 2004). Furthermore, proBNP was also found to be secreted in hESC-CMs exposed to mechanical strain (Ovchinnikova et al. 2018). proBNP was expressed in hESC-CMs cultured on laminin-coated glass coverslips and Matrigel-coated Aclar, both matrices with supraphysiological stiffness, after 14 days of culture (Fig. S2). Comparing laminin-coated glass coverslips with Matrigel-coated Aclar and PDMS coverslips after 35 days in culture showed no significant differences, although laminin-coated glass coverslips had the highest and Matrigel-coated PDMS had the lowest mean proBNP signal intensity. Expression of proBNP indicates mechanical stress in hESC-CMs grown on substrates of supraphysiological stiffness.

### Substrate-dependent effects on morphology of hESC-CMs

**Fig. 3:**
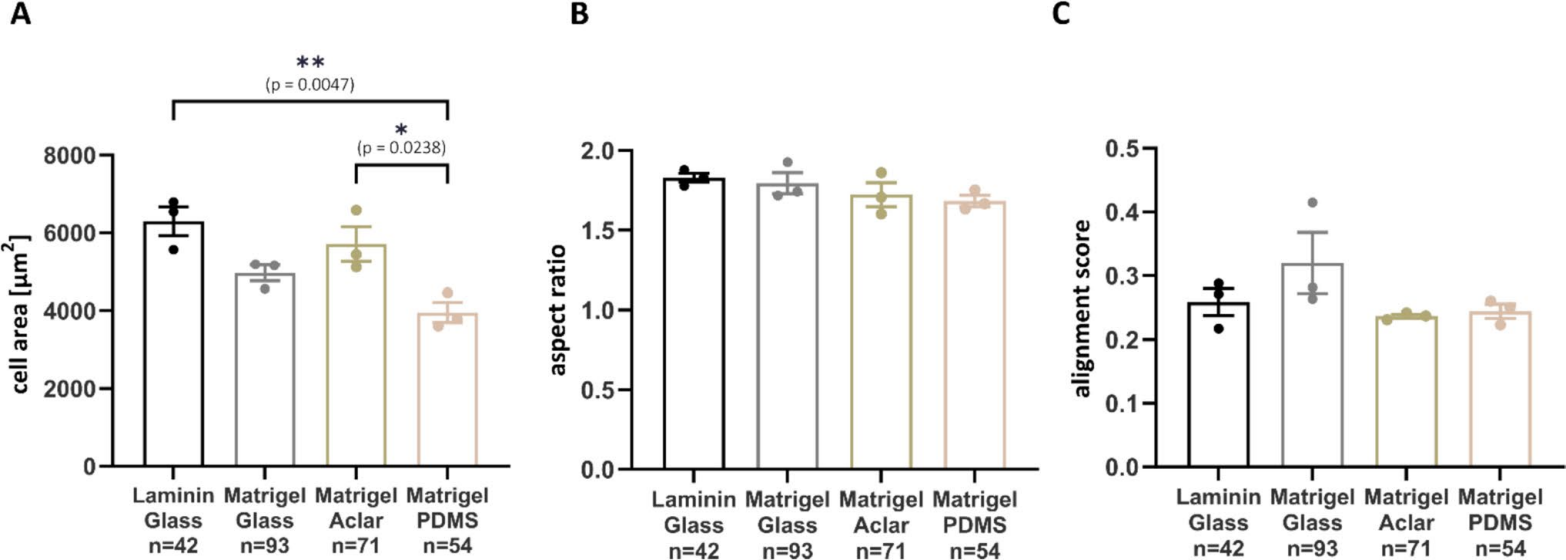
Morphological analysis of single hESC-CMs cultured on different substrates (surface and matrix combinations). **A**–**C** Analysis of cell area (**A**), aspect ratio (**B**), and myofibrillar alignment score (**C**) of hESC-CMs cultured on laminin-coated glass coverslips or Matrigel-coated glass, Aclar, and PDMS coverslips for 10 days. The values of all analyzed cells per coverslip were averaged. CMs from 3 coverslips of one differentiation. Mean ± SEM. *p < 0.05, **p < 0.01, one-way ANOVA with Tukey’s multiple comparisons test

To test whether substrate stiffness and coating also affects parameters of cell morphology, we assessed cell area, length-to-width ratio (aspect ratio), and myofibrillar alignment on different substrates. The highest mean cell area was observed on laminin-coated glass coverslips (6300 µm^2^), which was significantly larger than the mean cell area on Matrigel-coated PDMS (3954 µm^2^) coverslips (Fig. 3A). These findings indicate cellular hypertrophy on the more rigid matrix, which was also reflected by a significantly larger cell area on Matrigel-coated Aclar coverslips (5715 µm^2^) compared with Matrigel-coated PDMS. No significant differences in aspect ratio could be detected between hESC-CMs grown on different substrates, with the mean aspect ratio ranging from 1.68 to 1.83 (Fig. 3B). The myofibrillar alignment score was overall comparable between investigated substrates. The highest mean alignment score was observed in hESC-CMs cultured on Matrigel-coated glass (0.32; Fig. 3C).

### Effect of Ca^2+^ on MyHC and MLC2 isoform expression in single hESC-CMs

**Fig. 4:**
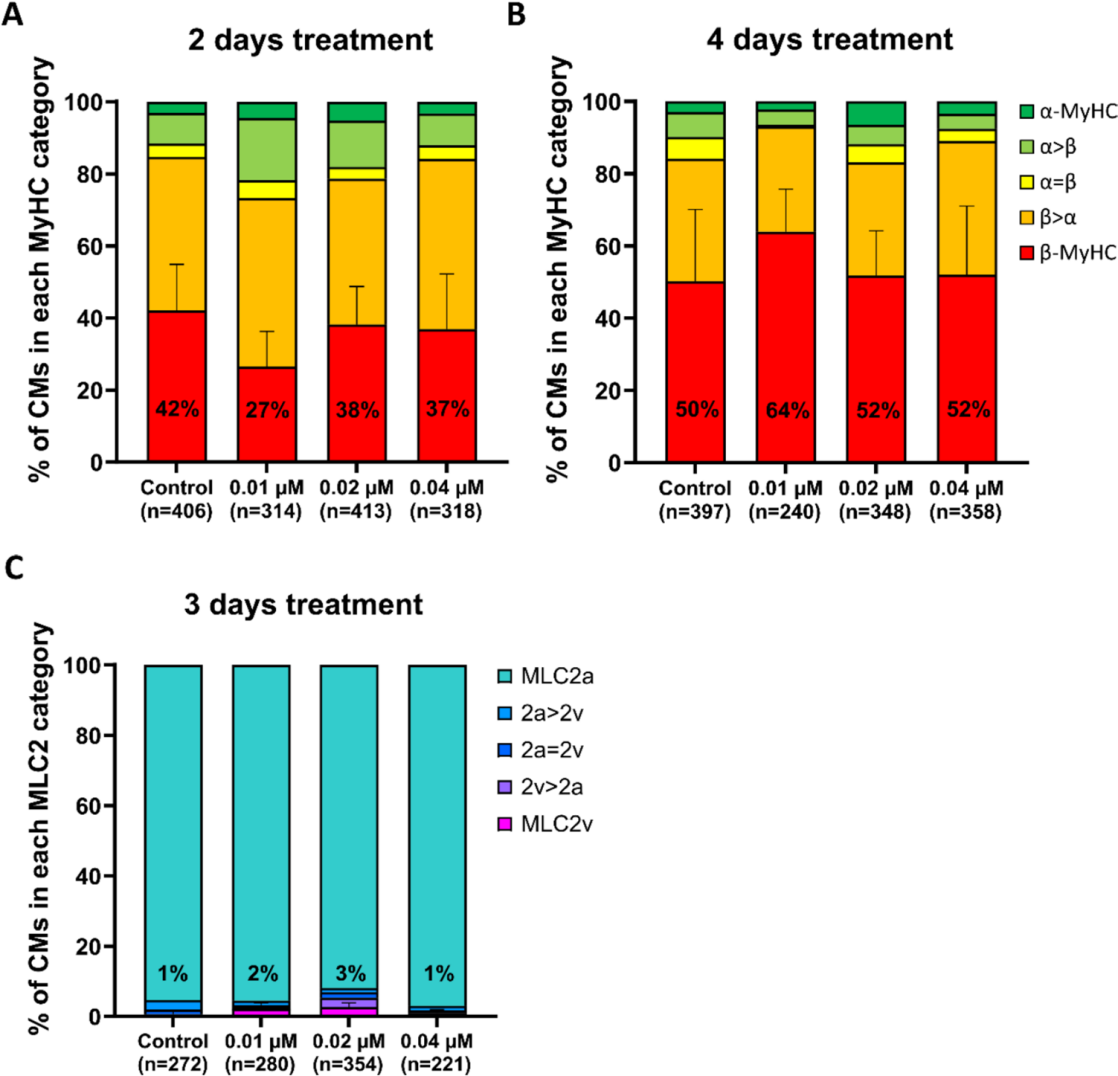
Analysis of the effect of Ca^2+^ ionophore A23187 on MyHC and MLC2 isoform expression in hESC-CMs cultured on laminin-coated glass coverslips. **A**–**C** Semiquantitative single cell IF analysis of MyHC and MLC2 isoform expression in hESC-CMs treated with 0.01 μM, 0.02 μM, or 0.04 μM Ca^2+^ ionophore A23187 for **A** 2, **B** 4, and **C** 3 days, from day 7 on using specific antibodies against **A**, **B** α- (green) and β- (red) MyHC or **C** MLC2a (cyan) and MLC2v (magenta). Nuclei were stained with DAPI (blue). The fractions of cells in the different categories (see Material and methods) are shown as percentage of the total number of cells analyzed (n, set to 100%). CMs from 3–4 coverslips of one differentiation, except for (**B**) control and 0.02 μM Ca^2+^ ionophore (5 coverslips of two differentiations). Mean ± SD. One-way ANOVA with Tukey’s multiple comparisons test was performed based on exclusively β-MyHC (red) and MLC2v (magenta), resp., expressing CMs only

Ca^2+^ has been implicated in mechanosensation/-transduction and related pathways (Sadoshima and Izumo 1997) as well as in intracellular signaling, e.g. in calcineurin-dependent cardiac hypertrophy (Molkentin et al. 1998), in cardiomyocytes. Furthermore, Ingenuity Pathways Analysis (IPA, Qiagen) of MyHC (*MYH6, MYH7*)- and MLC2 (*MYL2, MYL7*)-associated genes obtained from the *Gene* database of NCBI showed that calcium signaling is enriched in both *MYH6*/*MYH7* and *MYL2*/*MYL7* datasets (Fig. S3). To investigate a possible role of intracellular Ca^2+^ for expression of MyHC and MLC2 isoforms, hESC-CMs cultured on laminin-coated glass coverslips were treated with 3 different concentrations of Ca^2+^-ionophore A23187. The proportion of exclusively β-MyHC expressing hESC-CMs was not significantly different between hESC-CMs treated with 0.01, 0.02, or 0.04 µM ionophore for 2 or 4 days compared with control hESC-CMs (Fig. 4A, B). No changes in exclusively MLC2v expressing hESC-CMs could be observed after 3 days of Ca^2+^-ionophore treatment (Fig. 4C). 92–97% of hESC-CMs expressed exclusively MLC2a under all conditions. Taken together, experiments with Ca^2+^-ionophore do not provide evidence that changes of intracellular Ca^2+^ concentration are involved in the regulation of β-MyHC or of MLC2v expression at single-cell level in hESC-CMs cultured on laminin-coated glass coverslips.

### Effect of integrin-downstream signaling on MyHC expression

**Fig. 5:**
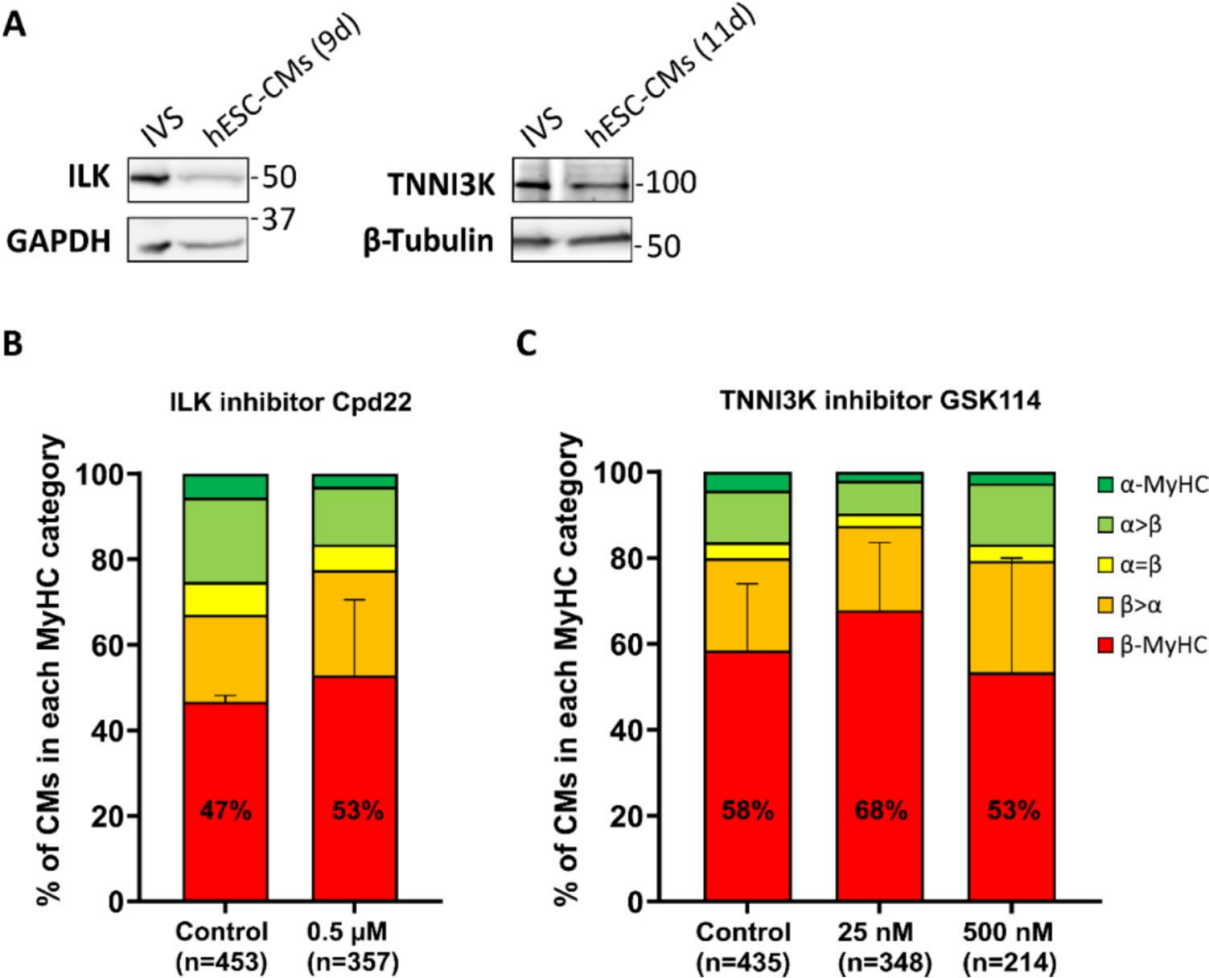
Analysis of ILK and TNNI3K inhibition on MyHC isoform expression in hESC-CMs cultured on laminin-coated glass coverslips. **A** Western blot analysis of ILK and TNNI3K expression in hESC-CMs cultured for 9 or 11 days as indicated. GAPDH, β-tubulin: loading controls. IVS: human interventricular septum sample. **B**, **C** Semiquantitative single cell IF analysis of MyHC isoform expression in hESC-CMs treated with **B** 0.5 µM of ILK inhibitor Cpd22 or with **C** 25 or 500 nM of TNNI3K inhibitor GSK 114 for 3 days, from day 7 on using specific antibodies against α- (green) and β- (red) MyHC. The fractions of cells in the different categories (see Material and methods) are shown as percentage of the total number of cells analyzed (n, set to 100%). CMs from 3 coverslips of one differentiation. Mean ± SD. Student’s t-test (Cpd22) and one-way ANOVA (GSK 114) were performed based on exclusively β-MyHC (red) expressing CMs only

To identify potential pathways which might underlie the regulation of myosin isoform expression, we used IPA of *MYH6*/*MYH7* and *MYL2*/*MYL7* NCBI gene datasets. IPA demonstrated that integrin-downstream mediator ILK signaling is enriched in canonical pathways based on *MYH6*/*MYH7*-associated dataset but not in *MYL2*/*MYL7* dataset (Fig. S3). We analyzed a possible role of ILK and of cardiac troponin I (cTnI)-interacting kinase (TNNI3K), a cardiac-specific kinase with sequence similarity to ILK (Zhao et al. 2003), for MyHC isoform expression in hESC-CMs. Western blotting demonstrated that both kinases are expressed in hESC-CMs (Fig. 5A). Neither treatment of hESC-CMs grown on laminin-coated glass coverslips for 3 days with 0.5 μM of the ILK inhibitor Cpd22 nor with 25 or 500 nM of the TNNI3K inhibitor GSK 114 from day 7 on led to significant changes in the proportion of exclusively β-MyHC expressing hESC-CMs (Fig. 5B, C). Treatment with 2 μM Cpd22 led to complete loss of all cells. Taken together, neither ILK nor TNNI3K have an impact on MyHC isoform expression on single-cell level in hESC-CMs cultured on laminin-coated glass coverslips.

In addition, treatment with a β1 integrin activating antibody neither led to further increases in exclusively β-MyHC expressing hESC-CMs (Fig. S4A) nor to further phosphorylation (activation) of integrin-downstream targets FAK and extracellular signal-regulated kinase (ERK) 1/2 (Fig. S4B). These data suggest an already high degree of integrin/FAK activation under the applied culture conditions on a stiff matrix.

### Epigenetic regulation of MyHC expression

**Fig. 6:**
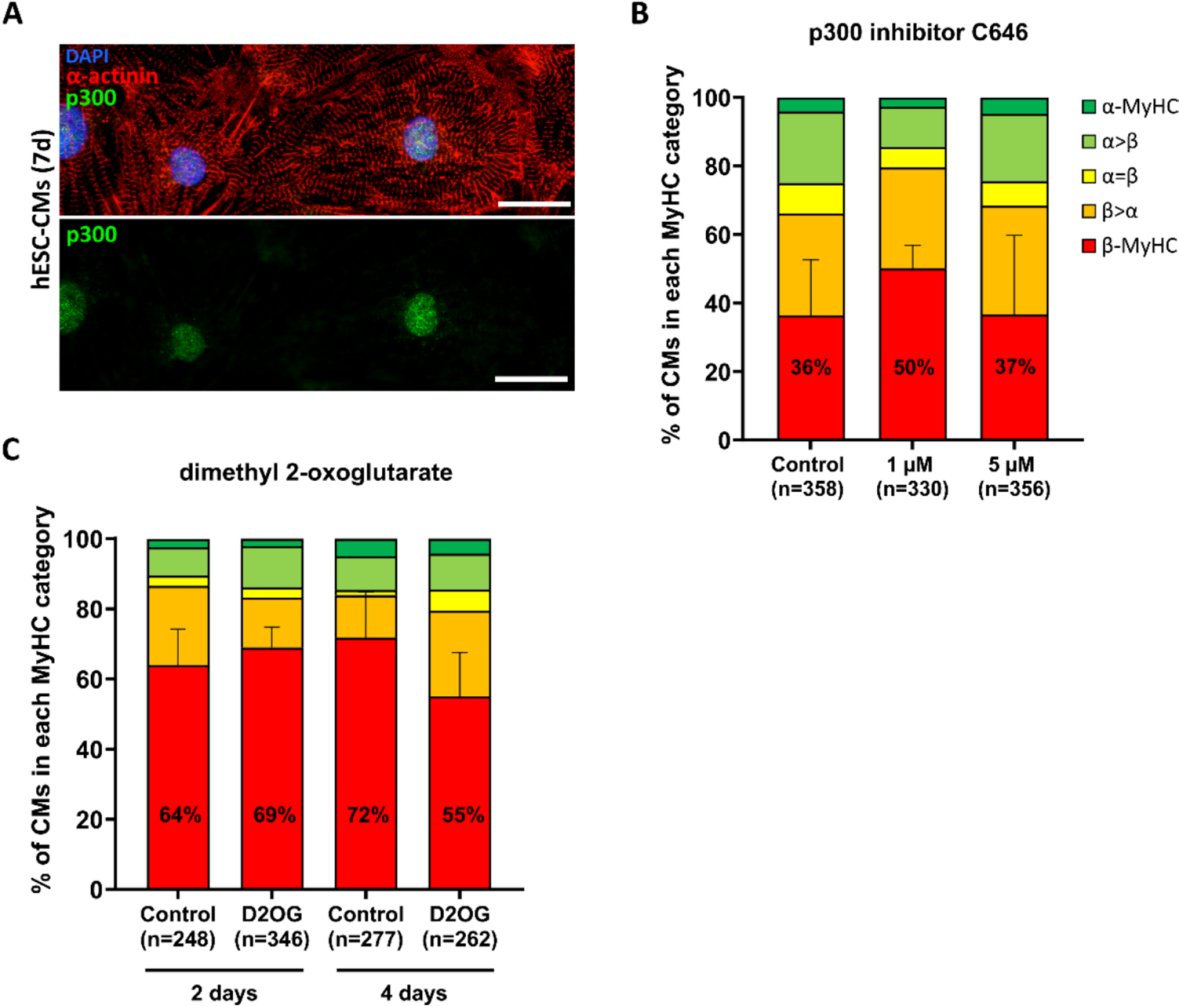
Analysis of epigenetic modulation on MyHC isoform expression in hESC-CMs cultured on laminin-coated glass coverslips. **A** Single cell IF analysis of p300 expression in hESC-CMs cultured for 7 days using specific antibodies against p300 (green) and against α-actinin (red) for identification of CMs. Nuclei were stained with DAPI (blue). Scale bars: 25 μm. **B**, **C** Semiquantitative single cell IF analysis of MyHC isoform expression in hESC-CMs treated with **B** 1 or 5 μM p300 inhibitor C646 for 2 days, or with **C** 4 mM membrane permeable 2-oxoglutarate derivate dimethyl 2-oxoglutarate (D2OG) for 2 or 4 days, from day 7 on. The fractions of cells in the different categories (see Material and methods) are shown as percentage of the total number of cells analyzed (n, set to 100%). CMs from 3 coverslips and one differentiation per group. Mean ± SD. One-way ANOVA (C646) and Student’s t-test (D2OG) were performed based on exclusively β-MyHC (red) expressing CMs only

It has been demonstrated that *MYH7* gene expression is also regulated by epigenetic modification such as acetylation (activation) and methylation (inhibition) (Meissner et al. 2011; Fang et al. 2016). A possible involvement of acetylation in *MYH7* expression in hESC-CMs was investigated on protein level using C646, a small molecule inhibitor of the histone acetyltransferase function of transcriptional co-activator p300 (Bowers et al. 2010). p300 was shown to be expressed in the nuclei of hESC-CMs cultured for 7 days on laminin-coated glass coverslips (Fig. 6A). 1 and 5 μM C646 did not induce statistically significant changes in the proportion of exclusively β-MyHC expressing hESC-CMs grown on laminin-coated glass coverslips treated for 3 days from day 7 on (Fig. 6B). The impact of methylation was analyzed using 2-oxoglutarate (2OG; α-ketoglutarate) as cosubstrate for dioxygenase TET2 to induce demethylation of promoter DNA (Wu and Zhang 2017). Treatment of hESC-CMs grown on laminin-coated glass coverslips with 4 mM of membrane-permeable 2OG derivative dimethyl 2-oxoglutarate (D2OG) for 2 and 4 days from day 7 on did not significantly change the relative amount of exclusively β-MyHC expressing hESC-CMs (Fig. 6C). To conclude, p300-dependent modulation of acetylation and 2OG-dependent regulation of methylation appears not to be involved in the regulation of β-MyHC expression at single-cell level in hESC-CMs cultured on laminin-coated glass coverslips.

## Discussion

We have previously shown that long-term culture of hESC-CMs on laminin-coated glass coverslips can efficiently promote β-MyHC expression at single-cell level (Weber et al. 2016). Interestingly, a switch from exclusively α- to exclusively β-MyHC expressing CMs in late vs. early cultures was also observed in different human induced pluripotent stem cell (hiPSC)-derived CM lines cultured on laminin-coated glass coverslips (data not shown). Furthermore, we could demonstrate upregulation of mechanosensation/-transduction-related pathways in hESC-CMs detached from their stiff matrix (laminin-coated glass coverslips) and subsequently replated on the same substrate (Osten et al. 2023). Reducing mechanical load by detaching cells led to re-expression of α-MyHC, while reintroducing an increased mechanical load through replating reconstituted the predominance of exclusively β-MyHC expressing hESC-CMs over time.

In the present investigations, no major differences in the proportion of exclusively β-MyHC expressing hESC-CMs were detected between different substrates (surface and matrix combinations) even in early stages of culture, indicating that surfaces with supraphysiological stiffnesses in combination with laminin are sufficient stimuli for predominant β-MyHC expression in hPSC-CMs. A major limitation of the presented study emerges from the fact that Young’s modulus of investigated surface materials is supraphysiological and is in part much higher than the reported ∼10 kPa of adult human myocardium (Pandey et al. 2018) that reaches up to 200–500 kPa at the end of diastole (Huyer et al. 2015). While the elastic modulus of matrix material Matrigel has been measured using atomic force microscopy (approximately 450 Pa) (Soofi et al. 2009), Young’s moduli of the investigated surface/matrix combinations were not determined so far. The high stiffness of the investigated surface materials is hypothesized to increase the mechanical load compared to cultivation as cardiac bodies in suspension, leading to increased mechanical stress. Indeed, IF analysis demonstrated expression of mechanical stress marker proBNP in hESC-CMs grown on stiff matrix. Taken together, increasing mechanical load by cultivating hESC-CMs on stiff matrix promotes expression of β-MyHC on single cell level.

Culture on laminin-coated glass coverslips resulted in cellular hypertrophy compared with less rigid substrates without affecting aspect ratio or myofibrillar alignment. Hypertrophy of hESC-CMs might be the result of profound mechanical stress on rigid substrates. Indeed, it has been demonstrated that stiffer substrates were associated with hypertrophic signaling (Querceto et al. 2022). In contrast, Herron et al. (Herron et al. 2016) showed a significantly larger size of human induced pluripotent stem cell-derived CMs on Matrigel-coated PDMS compared with cells grown on glass coated with Matrigel. The reason for this difference is not clear but might be related to different approaches to determine cell size (staining of cells with β-myosin vs. staining of cell borders with N-cadherin) or differences in cell confluency (subconfluent single cells vs. confluent monolayers). The aspect ratios of hESC-CMs grown on different substrates were considerably lower than those of adult human cardiomyocytes, in line with what has been shown before for hPSC-CMs (Denning et al. 2016), and with the non-adult character of the hESC-CMs, presumably aggravated by an early stage of culture.

It has been shown that substrate stiffness can also affect functional parameters. We have previously demonstrated faster twitch contraction and calcium transients in CMs grown on laminin-coated glass coverslips compared with culture as cardiac bodies (CB) in suspension (Weber et al. 2016). Studies with polyacrylamide (PAA) gels in the more physiological kPa stiffness range using a transgenic hESC-CM reporter line demonstrated significantly reduced shortening but increased contractile force under higher stiffness compared with physiological stiffness (Ribeiro et al. 2020). These findings are in line with previous findings from Hazeltine et al. (Hazeltine et al. 2012) showing that increased stiffness of PAA hydrogel substrates led to a stronger contraction force in rat neonatal CMs and hPSC-CMs but are in contrast to Wheelwright et al. (Wheelwright et al. 2018) demonstrating decreased total and normalized force as a function of increased substrate stiffness (PAA gels) in hiPSC-CMs. Furthermore, culture of hESC-CMs on rigid tissue culture plates (~ 3 GPa) has been shown to lead to increased beating frequency (Arshi et al. 2013).

In contrast to β-MyHC expression, MLC2v expression was partially promoted by Matrigel-coating. Interestingly, this only holds for Aclar and PDMS, but not for glass as surface material. In addition to stiffness, there are likely also differences in surface topography and roughness, which may influence cell adherence, cytoskeletal organization, and proliferation (Ross et al. 2012). Furthermore, it cannot be excluded that higher hydrophobicity of Aclar and PDMS in comparison with glass coverslips (Wu 1971; Mitra et al. 2016) may affect coating efficiency and cell attachment (Webb et al. 1998) on different surface materials at least to some degree. Matrigel is in contrast to laminin a multicomponent coating, containing different growth factors such as tumor growth factor-β (TGF-β), epidermal growth factor (EGF), insulin-like growth factor (IGF-1), and basic fibroblast growth factor (bFGF) (Vukicevic et al. 1992). The ECM is known to be a reservoir for growth factors (Rozario and DeSimone 2010), and supposed binding of these growth factors to surface receptors of hESC-CMs might activate growth factor-dependent pathways. Growth factor-dependent pathway activation has been shown to cross-talk with integrins, but also to alter cellular signaling and cytoskeletal organization independently of integrins (Ross 2004). Regarding the different effect of Matrigel coating on investigated surface materials on MLC2v expression, it is conceivable that MLC2v expression necessitates both mechanical and ECM ligand-dependent stimuli, possibly to activate integrins and initiate downstream signaling events.

The hESC-CMs used in this study were differentiated using a standard CM differentiation protocol based on biphasic Wnt pathway modulation in absence of retinoic acid (Schwanke et al. 2014; Weber et al. 2016; Osten et al. 2023; Kriedemann et al. 2024), which is reported to yield CMs of different subtypes, including ventricular CMs, while the addition of retinoic acid promotes atrial CM differentiation (Cyganek et al. 2018; Dark et al. 2023; Selvakumar et al. 2024). So far, despite detection of robust *MYL2* mRNA expression (encoding for MLC2v) at early time points in hPSC-CMs (data not shown), exclusively atrial MLC2a expressing hESC-CMs predominate after 35 days of culture on Matrigel-coated stiff surfaces, indicating the necessity for additional stimuli to obtain CMs co-expressing predominantly ventricular isoforms of myosin heavy and light chain. A possible approach may include more prolonged time in culture, as previously demonstrated for hiPSC-CMs cultured as embryoid bodies for up to 360 days (Kamakura et al. 2013), or modulation of the differentiation protocol to promote left ventricle-like CM differentiation e.g. by retinoic acid pathway blocking (Dark et al. 2023).

While integrin downstream mediator FAK promoted β-MyHC but not MLC2v expression in hESC-CMs (Osten et al. 2023), ILK and also TNNI3K had no effect on the proportion of exclusively β-MyHC expressing hESC-CMs. Both TNNI3K and ILK are highly expressed in cardiac muscle (Zhao et al. 2003; Bendig et al. 2006). ILK is not only activated by substrate adhesion but also by growth factors, phosphorylating downstream targets such as MLC2 and protein kinase B in vivo (Bendig et al. 2006). Studies on transgenic mice also suggest a significant role of ILK as a scaffolding/adapter protein, while its kinase activity appears not to be essential for mammalian development (Lange et al. 2009). Therefore, a possible role of ILK and TNNI3K for expression of MLC2 isoform needs to be further investigated.

In accordance with the absence of significant effects of Ca^2+^ ionophore, treatment of hESC-CMs with the calcineurin-inhibitor FK506 (100 nM) for 4 days had no effect on the proportion of exclusively β-MyHC cells (data not shown). Considering previously published data on Ca^2+^/calcineurin-induced *Myh7* expression in skeletal muscle C2C12 myotubes (Meissner et al. 2011), *MYH7* gene expression is evidently differentially regulated at least in hESC-CMs. Of note, Ca^2+^/calcineurin signaling has been shown to promote *Myh7* mRNA expression in mouse model of cardiac hypertrophy (Molkentin et al. 1998). We have not determined the intracellular Ca^2+^ concentrations after Ca^2+^ ionophore treatment. However, treatment of hESC-CMs with higher Ca^2+^ ionophore concentrations (0.1 μM, 0.5 μM) significantly reduced the number of intact cells (data not shown). Indeed, profound increases in intracellular Ca^2+^ concentration are well known to induce apoptosis (Harr and Distelhorst 2010). Overall, our data suggest that alterations of intracellular Ca^2+^ concentration are not involved in the regulation of β-MyHC or of MLC2v expression in hESC-CMs. It cannot be excluded that differences in calcium handling of hPSC-CMs and mature cardiomyocytes (Yang et al. 2014) contribute to our findings.

The presented data further indicate that *MYH7*/MyHC expression in hESC-CMs is not affected by epigenetic modifications involving p300 HAT or 2OG on single cell level, in case of p300 again in contrast to C2C12 myotubes (Meissner et al. 2011). Concerning acetylation, this finding might be substantiated by expanding the investigations to another HAT, CBP (CREB-binding protein), a transcriptional coactivator closely related to p300 (Dancy and Cole 2015), or by targeting other functional domains of p300, such as its bromodomain that has been implicated to play a role in cardiac reprogramming (Lim et al. 2021). Although TET2 plays an important role for demethylation, other demethylases might contribute to the regulation of *MYH7* gene expression (Das et al. 2023).

## Conclusions

In conclusion, our data demonstrate a different impact of substrates on the expression of adult ventricular markers β-MyHC vs. MLC2v in hESC-CMs at single cell level, indicating that expression of β-MyHC and MLC2v depends on different, possibly independent stimuli. These findings emphasize the complexity of maturation of hPSC-CMs and underscores the need to implement multiple stimuli for the promotion of a comprehensive adult ventricular phenotype.

## Supplementary Information

Below is the link to the electronic supplementary material.

Supplementary file1 (DOCX 2749 KB)

## Data Availability

No datasets were generated or analysed during the current study.
