# Supplementary material for: Differential impact of substrates on myosin heavy and light chain expression in human stem cell-derived cardiomyocytes at single-cell level
Source: J Muscle Res Cell Motil. 2025 Feb 13;46(2):119–33. doi: 10.1007/s10974-025-09690-2 (PMC12680726; doi:10.1007/s10974-025-09690-2)
Supplement: Supplementary file 1 — Supplementary file1 (DOCX 2749 KB) [file 10974_2025_9690_MOESM1_ESM.docx]

**Supplementary Information**

**Differential impact of substrates on myosin heavy and light chain expression in human stem cell-derived cardiomyocytes at single-cell level**

Journal of Muscle Research and Cell Motility

Felix Osten^1^ · Alea K. Bodenschatz^1^ · Karina Ivaskevica^1^ · Simon Kröhn^1,*^ · Birgit Piep^1^ · Tim Holler^1^ · Jana Teske^2^ · Judith Montag^1,+^ · Bogdan Iorga^1,3^ · Natalie Weber^1, #^ · Robert Zweigerdt^2^ · Theresia Kraft^1^ · Joachim D. Meissner^1^

^1^Institute of Molecular and Cell Physiology, Hannover Medical School, Hannover, Germany ^2^Leibniz Research Laboratories for Biotechnology and Artificial Organs (LEBAO), Department of Cardiothoracic, Transplantation and Vascular Surgery, Hannover Medical School, Hannover, Germany

^3^Department of Analytical Chemistry and Physical Chemistry, Faculty of Chemistry, University of Bucharest, Bucharest, Romania

^*^, present address: Department of Nuclear Medicine, Hannover Medical School, Hannover

^+^, present address: Faculty of Medicine, MSB Medical School Berlin, Berlin

#, present address: Institute of Molecular and Translational Therapeutic Strategies (IMTTS), Hannover Medical School, Hannover, Germany

Corresponding author: Felix Osten

[osten.felix@mh-hannover.de](mailto:osten.felix@mh-hannover.de)

**
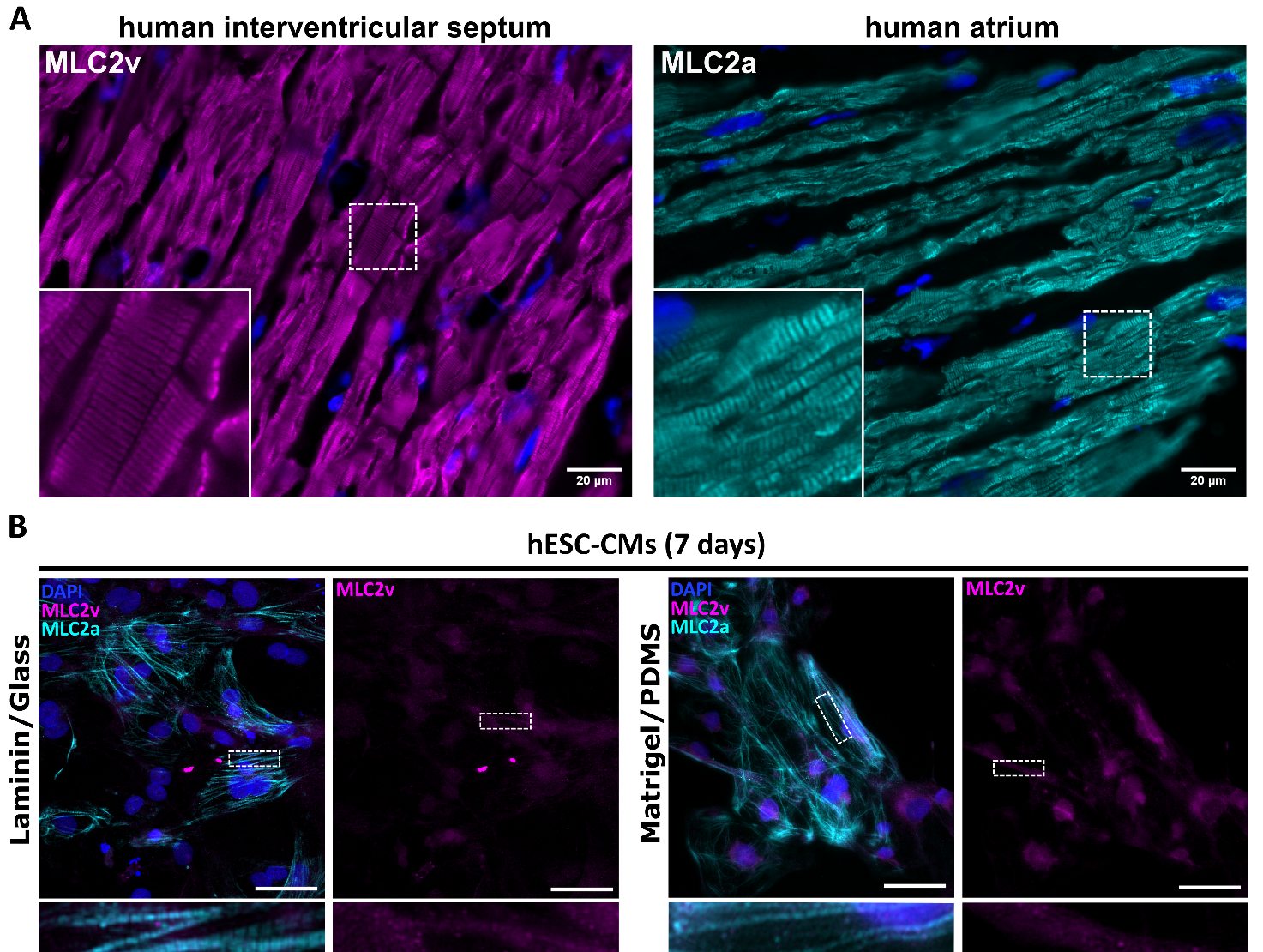
**

**Fig. S1** MLC2v and MLC2a expression in adult human cardiac tissue sections and in hESC-CMs cultured for 7d. (A) Immunofluorescence stainings of human interventricular septum (left) and human atrium (right) cryosections with specific antibodies against MLC2v (magenta, left) and MLC2a (cyan, right), with DAPI (blue) as nuclear counterstain. The insets (bottom left in each image) show magnified views of sarcomeric striation patterns in the indicated regions. Scale bars: 20 µm. (B) Immunofluorescence stainings of hESC-CMs cultured for 7 days on laminin-coated glass coverslips (left) and Matrigel-coated PDMS (right) with specific antibodies against MLC2v (magenta) and MLC2a (cyan), with DAPI (blue) as nuclear counterstain. Magnified views of the indicated regions below each image demonstrate sarcomere-specific striations for MLC2a and only unspecific signal for MLC2v. Scale bars: 50 µm.


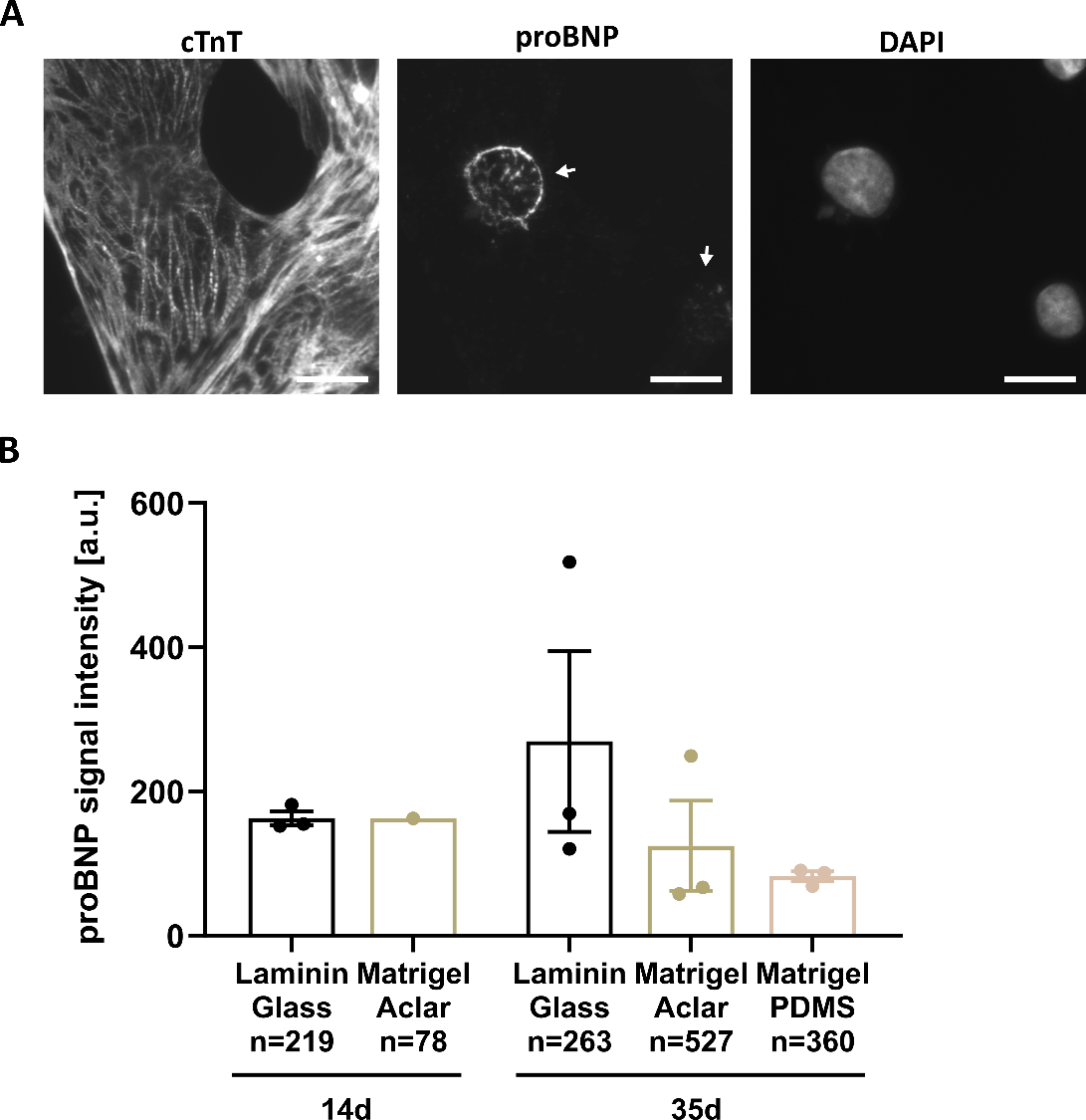


**Fig. S2** Analysis of proBNP isoform expression in single hESC-CMs cultured on different substrates (surface and matrix combinations). (A) Representative images showing single cell IF analysis of proBNP expression in hESC-CMs cultured for 35d on Matrigel-coated Aclar using specific antibodies against proBNP and against cardiac troponin T (cTnT) for identification of CMs. Nuclei were stained with DAPI. White arrows depict perinuclear area of CMs with and without proBNP signal. Scale bars: 20 μm. (B) Semiquantitative single cell IF analysis of proBNP expression in hESC-CMs cultured for 14 or 35d on laminin-coated glass coverslips, or Matrigel-coated Aclar or PDMS as indicated. ProBNP signal intensity (a.u., arbitrary units) was analyzed in the nuclear and perinuclear (arrows in (A)) regions of single CMs and the values of all analyzed cells per coverslip were averaged. CMs from 1-3 coverslips and differentiations, respectively. Mean ± SEM. One-way ANOVA with Tukey’s multiple comparisons test resulted in no significant differences between means.


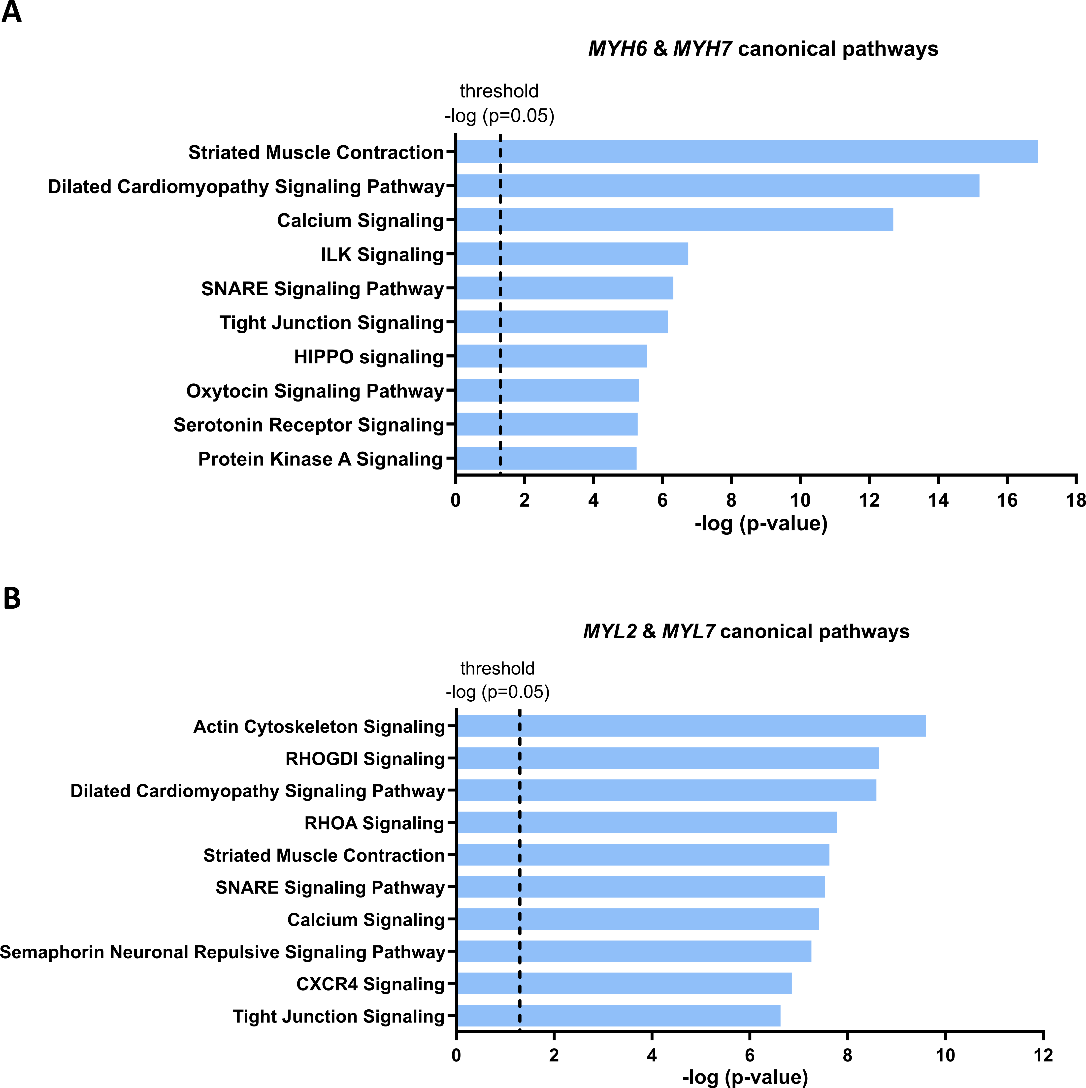


**Fig. S3** Ingenuity Pathway Analysis showing predicted canonical pathways based on NCBI gene datasets obtained from *MYH6* and *MYH7* or *MYL2* and *MYL7* search queries. Ten highest ranked predicted canonical pathways according to NCBI database of (A) *MYH6* and *MYH7* and of (B) *MYL2* and *MYL7* associated genes. Results are sorted by -log p-value ranking, and the p-values of overlap for canonical pathways were calculated by right-tailed Fisher’s exact test.


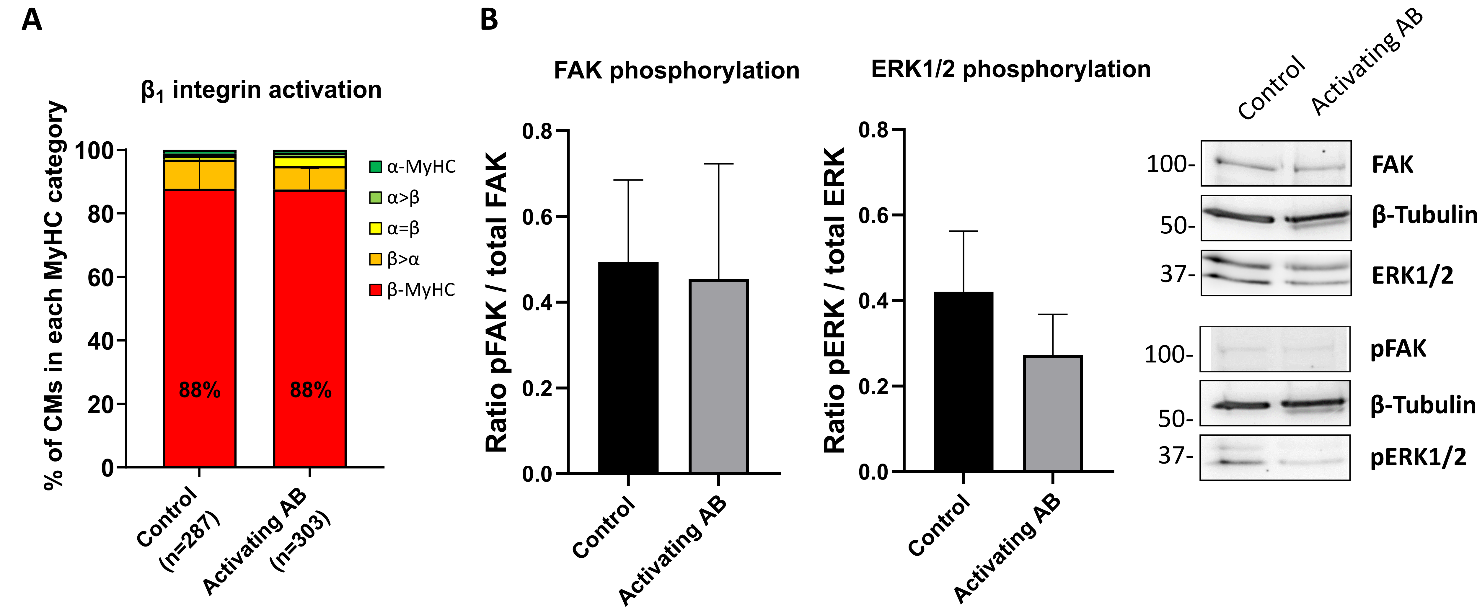


**Fig. S4** Effect of β_1_ integrin activation on MyHC expression and FAK and ERK1/2 phosphorylation in hESC-CMs cultured for 7 days on laminin-coated glass coverslips and treated with β1 integrin activating antibody for 4 days (Activating AB). (A) Semiquantitative single-cell IF analysis of MyHC isoform expression in single hESC-CMs. The fractions of cells in the different categories (see Material and methods) are shown as percentage of the total number of cells analyzed (n, set to 100%). CMs from 3 coverslips of one differentiation. Student’s t-test was performed based on exclusively β-MyHC (red) expressing CMs only. (B) Western Blot analysis of FAK, phospho-FAK (pFAK), ERK1/2 and phospho-ERK1/2 (pERK1/2) expression. The ratio of pFAK/FAK and pERK1/2/ERK1/2 expression was determined densitometrically. Mean ± SEM; n = 3 individual coverslips from one differentiation. Representative Western Blot; loading control: β-tubulin.

**Table S1** Analysis of MyHC isoform expression in single hESC-CMs cultured on different substrates (surface and matrix combinations) for 7 and 10 days.

|  | **7 days** | |  | **10 days** | |
| --- | --- | --- | --- | --- | --- |
| **Tukey's multiple comparisons test** | **Adjusted p-value** | **Summary** |  | **Adjusted p-value** | **Summary** |
| Laminin/Glass vs. Laminin/PDMS | 0.0713 | ns |  | 0.9898 | ns |
| **Laminin/Glass vs. Laminin/Aclar** | **0.0499** | ***** |  | 0.9999 | ns |
| Laminin/Glass vs. Matrigel/Glass | 0.8277 | ns |  | 0.6988 | ns |
| Laminin/Glass vs. Matrigel/PDMS | 0.2360 | ns |  | 0.1647 | ns |
| Laminin/Glass vs. Matrigel/Aclar | 0.6411 | ns |  | 0.3109 | ns |
| Laminin/PDMS vs. Laminin/Aclar | >0.9999 | ns |  | 0.9989 | ns |
| Laminin/PDMS vs. Matrigel/Glass | 0.4238 | ns |  | 0.9492 | ns |
| Laminin/PDMS vs. Matrigel/PDMS | 0.9687 | ns |  | 0.3795 | ns |
| Laminin/PDMS vs. Matrigel/Aclar | 0.6168 | ns |  | 0.6162 | ns |
| Laminin/Aclar vs. Matrigel/Glass | 0.3226 | ns |  | 0.8168 | ns |
| Laminin/Aclar vs. Matrigel/PDMS | 0.9164 | ns |  | 0.2305 | ns |
| Laminin/Aclar vs. Matrigel/Aclar | 0.4955 | ns |  | 0.4150 | ns |
| Matrigel/Glass vs. Matrigel/PDMS | 0.8381 | ns |  | 0.8412 | ns |
| Matrigel/Glass vs. Matrigel/Aclar | 0.9991 | ns |  | 0.9734 | ns |
| Matrigel/PDMS vs. Matrigel/Aclar | 0.9559 | ns |  | 0.9974 | ns |

Overview of one-way ANOVA results with Tukey’s multiple comparisons test of data presented in Figure 1, based on exclusively β-MyHC expressing hESC-CMs. *, p<0.05; ns: not significant. Significant results are highlighted in bold letters.
